# Honey Bee Queen Replacement: An Analysis of Changes in the Preferences of Polish Beekeepers through Decades

**DOI:** 10.3390/insects11080544

**Published:** 2020-08-17

**Authors:** Małgorzata Bieńkowska, Aleksandra Łoś, Paweł Węgrzynowicz

**Affiliations:** Apiculture Division, Research Institute of Horticulture, 24–100 Puławy, Poland; malgorzata.bienkowska@inhort.pl (M.B.); pawel.wegrzynowicz@inhort.pl (P.W.)

**Keywords:** *Apis mellifera*, apiary management, apiculture, bee breeding, beekeeping trends, bee selection, monitoring, requeening, survey

## Abstract

**Simple Summary:**

In Poland, there are 1.68 million honey bee (*Apis mellifera*) colonies. We conducted a survey on honey bee (*Apis mellifera*) queen management. In the years 1980–2018, questionnaires were sent to beekeepers all over Poland. Questions regarding queen management throughout a single beekeeping season were asked each year: (1) the number of spring bee colonies with which the new season began, (2) the number of queens replaced throughout the season, and (3) the number of newly purchased queens. In total, 2964 questionnaires were selected. We examined the trends by decade timeslot, apiary size, and geographical location. In Poland, it is recommended to replace at least 30% of queens every year. Regardless of the decade and the size of the apiary, on average, above 90% of Polish beekeepers replace old queens with new ones in their colonies. In general, during the observed period, beekeepers replaced almost 52% of their queens, 21% of which were purchased. The involvement of purchased queens in colony management is associated with the size of the apiary, and it significantly grows with the number of colonies in the apiary. The percentage of purchased queens went up in all the voivodeships over time.

**Abstract:**

We conducted a survey on honey bee (*Apis mellifera*) queen management. Data were collected every year from 1980 to 2018. In total, 2964 questionnaires were collected from all over Poland. We examined the trends by decade timeslot, apiary size, and geographical location. Regardless of the decade and the size of the apiary, on average, above 90% of Polish beekeepers replace old queens with new ones in their colonies. In general, during the observed period, beekeepers replaced almost 52% of their queens, 21% of which were purchased. In the last decade, there was an upward trend in the percentage of beekeepers replacing queens throughout the country. The involvement of purchased queens in colony management is associated with the size of the apiary, and it significantly grows with the number of colonies in the apiary. The percentage of purchased queens went up in all the voivodeships over time. Research and education in this area are needed in order to track the trends and further improve Polish beekeepers’ practices.

## 1. Introduction

In Poland, there are 1.68 million honey bee (*Apis mellifera*) colonies. Currently, almost 30% of Polish apiaries comprise 21 to 50 colonies, and they are managed mainly by 51–65-year-old beekeepers. Professional apiaries (with over 80 colonies) constitute 3.7% of all apiaries and comprise 84,000 colonies in total [1]. In Poland, the law allows four subspecies to be bred: *Apis mellifera mellifera*, *A. mellifera carnica*, *A. mellifera caucasica*, and *A. mellifera ligustica*. There are three types of breeding programs in Poland: (1) genetic improvements of all subspecies, (2) cross-breeding programs, and (3) a national conservation program for *A. mellifera mellifera* and one line of *A. mellifera carnica*. *Apis mellifera carnica* is currently the most popular subspecies with Polish bee breeders and beekeepers. In 2017, Polish bee breeders produced over 180,000 *A. mellifera carnica* queens, representing 95% of all the queens reared in breeding apiaries [2]. High-quality honey bee queens are the most basic and key element of profitable apiaries [3]. Nevertheless, unsolvable queen problems may still occur. This is believed to be one of the reasons for colony losses in Europe [4,5]. According to van der Zee et al. [6], overall loss rates for the winter of 2012–2013 for beekeepers with 1 to 50 colonies reached 20.9%, and for apiaries with 51 to 150 colonies, they reached 14.6% in Poland. Semkiw et al. [1] and Gray et al. [5] noted that in 2017/18 colony losses in Poland reached 14.2%, of which losses resulting from unsolvable queen problems were estimated at 3.5%. The cause-and-effect relationship is straightforward: The younger and more reproductive the queen is, the higher the number of workers and the greater the colony productivity are [2,7]. The consequences of neglecting the rejuvenation of queen populations by beekeepers may be severe, e.g., low colony productivity, a decrease in colony strength and disease resistance, swarming, overwintering failures, and colony losses, as well as more work and less work comfort [8,9,10,11,12,13]. On this account, the replacement of old, less and less fertile queens with new and high-quality ones should be treated as one of the essential beekeeping measures. All of these fundaments of sustainable and economically profitable beekeeping and queen replacement have been emphasized by scientists for years and are also fairly well received by beekeepers. Scientists have been engaged in extensive educational activities aimed at raising the awareness of importance of queen replacement and its meaning for colonies (for examples, see Reference [14,15,16,17]). Additionally, Polish beekeepers are encouraged to improve their practices, among other things, during international meetings and workshops, e.g., the Polish–German beekeeping conferences in 1995–2000 and the Polish–German SMARTBEES conference in 2018 [18]. Moreover, Polish beekeepers actively participated in the GEI experiment (which was a part of the COST action COLOSS) and EurBeSt activities [19]. Since the 1970s, it has been constantly suggested to beekeepers in Poland to replace queens every other year and recommended to include 30% of colonies in the apiary in each replacement management exercise [20]. New queens can be self-reared by beekeepers and/or purchased from breeders, e.g., those carrying out honeybee genetic improvement programs supervised by the National Animal Husbandry Centre [2,4,9]. However, access to information, awareness, and trends in apiary management have changed throughout the decades. The aim of the study was to investigate how this recommendation was actually applied in beekeeping practice in Poland, from 1980 to 2018.

## 2. Materials and Methods

### 2.1. Survey Design and Response Rate

In the years 1980–2018, questionnaires were sent to beekeepers all over Poland. The questionnaire was designed in the 1960s, and it was constantly improved by researchers from the Research Institute of Horticulture, until it assumed its current form, which was implemented in the 1980s. Hard copies of 1500 questionnaires were sent every year to several beekeepers’ associations all over Poland and to individual beekeepers who had declared their cooperation with the Research Institute of Horticulture. It was at the discretion of the respondents to complete the questionnaire and send back individually collected results and observations to the address of the Apiculture Division of the Research Institute of Horticulture. A total of 614 beekeepers took part in the survey. Some of them sent answers only once during the period under examination, and some responded repeatedly over successive years. Questionnaires in which beekeepers responded to the questions covered by this publication were selected for analyses. Questions regarding queen management throughout a single beekeeping season were asked each year: (1) the number of spring bee colonies with which the new season began, (2) the number of queens replaced throughout the season, and (3) the number of newly purchased queens. In total, 2964 questionnaires were selected. The number of the questionnaires is presented in Table 1.

The data were compiled and analyzed in terms of the following:

1. Trends by decade timeslot: (I) 1980–1989; (II) 1990–1999, (III) 2000–2009, and (IV) 2010–2018.
2. Apiary size, as follows:
  - Micro-apiaries, from 1 to 10 colonies;
  - Small apiaries, from 11 to 20 colonies;
  - Medium apiaries, from 21 to 50 colonies;
  - Big apiaries, from 51 to 80 colonies;
  - Professional apiaries, above 81 colonies.
3. Geographical location according to the administrative division of Poland (16 voivodeships).

### 2.2. Statistical Analysis

The data were analyzed for the three classifiers mentioned above. Interaction effects were also considered. In order to avoid underestimation, due to the low data frequency, triple “timeslot × apiary size × voivodeship” interactions were omitted from the analyses. The analyses also omitted cases in which less than 5 questionnaires were received (voivodeship: Dolnośląskie, Śląskie, and Warmińsko-Mazurskie × timeslot: 2010–2018).

**Figure 1:**
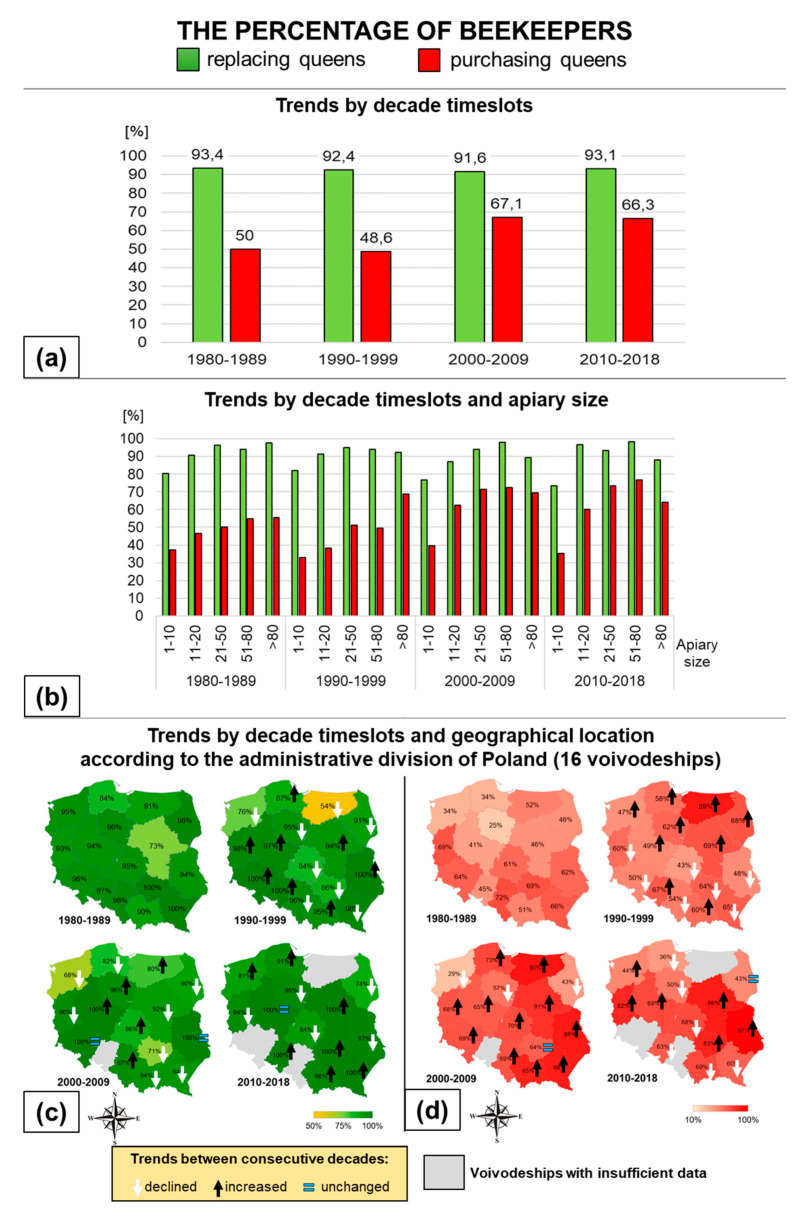
Percentage of beekeepers replacing queens, and percentage of beekeepers purchasing queens with regard only to beekeepers who replaced queens: (**a**) trends by decade timeslots; (**b**) trends by decade timeslot and apiary size; (**c**) trends by decade timeslot and geographical location for beekeepers replacing queens; (**d**) trends by decade timeslot and geographical location for beekeepers purchasing queens.

**Figure 2:**
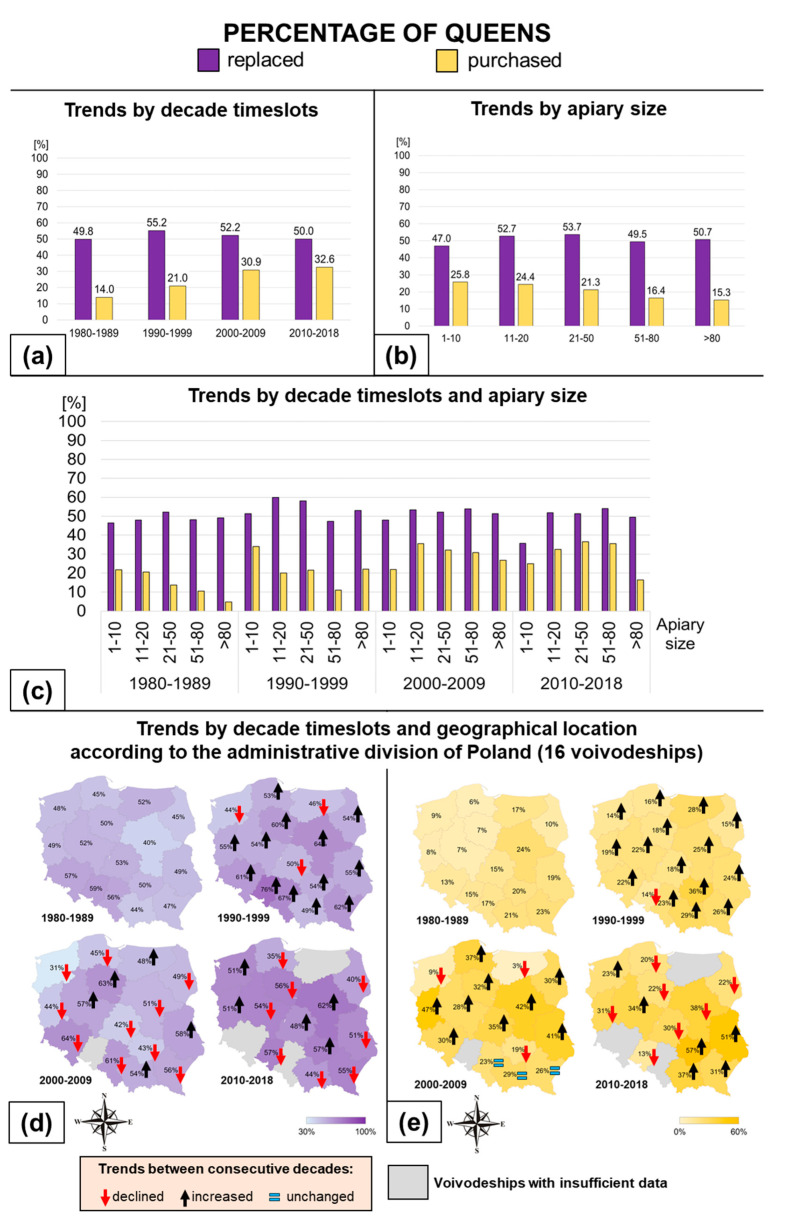
Percentage of replaced queens, and percentage of purchased queens in regard only to colonies in which queens were replaced: (**a**) trends by decade timeslot; (**b**) trends by apiary size; (**c**) trends by decade timeslot and apiary size; (**d**) trends by decade timeslot and geographical location for replaced queens; and (**e**) trends by decade timeslot and geographical location for purchased queens.

In the first stage of the statistical analysis, the distribution of the variables being examined was evaluated, using the Kolmogorov–Smirnov test. Due to the fact that the traits were non-normally distributed, generalized linear models (GLMs) were used for the analysis of the data. The numbers of beekeepers replacing and purchasing queens were treated as binominal variables and modeled by using GLMs with a logistic link function. All the models were built as a model with a baseline level, which was as follows: for timeslots 1980–1989, for apiary size micro-apiaries, and for voivodeship Dolnośląskie. The numbers of replaced and purchased queens were analyzed by means of GLMs, with a logarithmic link function and normal and Poisson noise distribution functions, respectively. The Wald test was used to test significance of the effects. The data in the Appendix A (Table A1, Table A2, Table A3 and Table A4) and in Figure 1a–d and Figure 2a–e are presented as percentages. Calculations and analyses were made by using STATISTICA v. 13 (Dell Inc., Austin, TX, USA, 2016).

## 3. Results

### 3.1. Beekeepers’ Preferences and Apiary Management Style

Regardless of the decade and the size of the apiary, on average, over 90% of Polish beekeepers replace queens in their colonies. On average, 54% of Polish beekeepers use purchased queens in apiary management. The percentage of beekeepers who purchased queens was the lowest in the 1990s and the highest in the 2000–2009 timeslot, i.e., 49% and 69%, respectively (Figure 1a; Table A1). In all the decades, the greater the size of the apiary, the higher the percentage of beekeepers who purchased queens. From decade to decade, more and more beekeepers with 21–50 colonies bought queens (Figure 1b; Table A1). In Southwestern and Southern Poland, a large percentage of beekeepers regularly replaced queens. The percentage of beekeepers replacing their queens fluctuated in Central and Northern Poland. In the last decade, there was an upward trend in the percentage of beekeepers replacing queens throughout the country (Figure 1c). Up to the third decade, the percentage of beekeepers purchasing queens increased steadily, and it levelled out throughout Poland between 2010 and 2018 (Figure 1d). The interactions between the values of the parameters being analyzed for beekeepers are presented in Table 2.

In Poland some beekeepers declared replacing all the queens (x = 100%) in their apiaries annually and some did not replace queens at all (x = 0%) (Table 3 and Table A2). Most often such a phenomenon can be observed in the smallest apiaries of 1 to 10 colonies, where 15.2% of beekeepers replaced all the queens and 21.5% of beekeepers did not replace any (Table 3).

### 3.2. Honey Bee Queen Replacement

In general, during the observed period, beekeepers replaced almost 52% of their queens, 21% of which were purchased. The percentage of the replaced queens increased significantly in the 1990s, to 55%, after which it steadily decreased to 50% in the following timeslots. From decade to decade, the percentage of purchased queens increased from 13% in the 1980s to 33% in 2010 and levelled out (Figure 2a,c; Table A3). The involvement of purchased queens in colony management is associated with the size of the apiary, and it increases significantly with the number of colonies in the apiary (this tendency is displayed by apiaries with no more than 80 colonies). Most of the queens, over 52%, were replaced in apiaries with 11–20 and 21–50 colonies (Figure 2b,c; Table A4). The percentage of replaced queens is evenly distributed throughout Poland. Since the 1980s and 1990s, the percentage of purchased queens increased in all the voivodeships. In Western Poland, in the Lubuskie voivodeship, the percentage of purchased queens increased from 7.9% in 1980–1989 to 47.3% in 2000–2009, and 30.8% in the last decade; in the Zachodnio-Pomorskie voivodeship, it increased from 8.5% in 1980–1989 to 23.3% in 2010–2018. In Eastern Poland, the Lubelskie voivodeship saw an increase in purchased queens from 19.4% (1980–1989) to 50.5% (2010–2018), with a consistently high percentage of replaced queens, and a similar situation was observed in the south of Poland, in the Podkarpackie voivodeship, with an increase from 22.7% to 31% (Figure 2d,e). The interactions between the values of the parameters being analyzed for queens are presented in Table 4.

## 4. Discussion

The replacement of queens in apiaries suggests beekeepers care for their stock and profits. According to scientific knowledge, poor-quality queens are a main management concern [3,13]. Various pathologies are prevalent in older queens rather than in young, newly mated ones [21]. Therefore, replacement of queens is one of the basic elements of proper apiary management. It enables an increase in their productivity [5,10,11,12,13]. In Poland, it is recommended to replace at least 30% of queens every year [20]. However, there are always (and will always be) apiaries which do not replace queens at all or replace even 100% of them (Table 1 and Table A2).

The origin of replaced queens can vary. Beekeepers look for a resource of queens in their own apiaries and employ queens from supersedure, swarms, and their own rearings. In this way, however, an undesirable limitation of the gene pool may occur within the apiaries. Therefore, the issue of sustainable queen replacement, including purchased queens, is so important (References [4,10] and personal observations). Purchased queens may come from various sources. According to Bieńkowska et al. [2], 68% of purchased queens originated from bee breeders, whereas the rest were of unknown origin. Similarly, Mutinelli et al. [22] claimed that international bee trade is not always officially registered, which is the main source of worldwide disease-dissemination risk. Additionally, Muñoz et al. [23] noticed that beekeeping practices such as the importation of non-native honey bee queens may interact with the conservation of honey bee biodiversity. Therefore, following Büchler et al. [4], it is worth highlighting that the conservation of honey bee diversity and the support for local breeding activities must be prioritized in order to prevent colony losses, optimize sustainable productivity, and enable continuous adaptation to environmental changes. Moreover, it was repeatedly stated that colonies with local-origin queens survive longer than those with non-local ones [4,24].

According to Gromisz [20], in 1967–1970, more than 90% of Polish beekeepers replaced queens in their apiaries, and over 33% of queens for the replacement were purchased. The replacement trend remains stable to this day, while the percentage of beekeepers purchasing queens has changed over time and has increased significantly from decade to decade; and over the last forty years, on average, 54% of beekeepers purchased queens (Figure 1a; Table A1). Historical data showed that purchased queens allowed for the rejuvenation of 8% of Polish resources [20], whereas, now, purchased queens satisfy more than 20% of beekeepers’ demand (Table A3). In comparison, Șeker et al. [25] showed that 57% of Turkish beekeepers replaced queens every other year, and almost 84% of them were bought (did not come from their own breeding).

According to Chauzat et al. [26], there is high heterogeneity in the apicultural industry within the European Union. The high proportion of non-professional beekeepers and the small number of colonies per beekeeper are common characteristics at the European level. In Poland, the replace and purchase rates are influenced by the size of the apiary. Queen-replacement activity is the most intense in medium apiaries, with 21–50 colonies (Figure 1b; Table A4). This can be explained by the fact that owners of small apiaries are more open to change and willing to experiment, even involving all of their colonies. Amateur beekeepers are highly active in introducing new queens, but they lack the caution that characterizes professional apiaries with more than 80 colonies. Owners of larger apiaries are more willing to invest in their own queens’ rearings; they want certainty and stability of value in regard to the queens’ performance qualities (References [20,26] and personal observations).

Döke et al. [27] claimed that overwintering success of colonies is influenced by the weight and population size reached by the colonies before winter rather than by their geographical origin. However, location determines apiary management style, and it can be of great importance to the colonies’ survival. The percentage of the replaced and purchased queens is geographically diverse (Figure 1c,d and Figure 2d,e). In the east of Poland (Podlaskie voivodeship), replacement focusing on swarms has survived, and bees are treated in a more traditional way than in the other regions. Other reasons have shaped the results for the northwest of Poland (Zachodnio-Pomorskie voivodeship), where 31–51% of queens were replaced, but purchased queens represented only 8% in the 1980s and up to 23% in the last decade. The low former rate of purchase was the result of a lack of access to breeding materials, as they must have been bought personally, and no breeding apiaries were present in that region then. The current increase was caused by the growth of mail-order sales of queens from beekeeping centers located in other regions. A different situation is observed in Middle Eastern Poland (Lubelskie voivodeship), where the percentage of replaced queens was always high, between 49 and 58%, and the percentage of purchased ones increased, from decade to decade, from 19% to 51%. This is due to the number of registered breeding apiaries and beekeeping centers in the region and the sustained possibility of direct, easy access to high quality queens. Apiary management style in Poland has changed from extensive to intensive, specialized, and profit-oriented. This could have been attributed to growing access to information and the political transformation of the country, all of which together raised the economic awareness of beekeepers. In the past, the level of queen replacement was mainly driven by the difficulties encountered by beekeepers in supplying themselves with high-quality material. The percentage of queen replacement in an apiary may be characterized by the extent to which unsolvable queen problems have been overcome, and thus the beekeeper’s commitment to improving the stock.

De la Rúa et al. [28] stated that trading in queens, combined with their promiscuous mating system, has exposed native European honey bees to increasing hybridization with non-local subspecies. This could lead to the loss of valuable combinations of traits shaped by natural selection. According to Plate et al. [9], even when unwanted admixtures of subspecies can be excluded in natural mating incidences, controlled mating is imperative for successful breeding efforts. Moreover, Pérez-Sato et al. [29] showed that multilevel selection can significantly improve the success of honey bee breeding programs. It is also worth mentioning that, in 2019, the National Breeding Association was created to further improve and develop the existing good breeding practices in Poland [30]. Following De la Rúa et al. [28], it is still worth emphasizing that international initiatives to collect data in order to study and evaluate beekeeping management and honey bee stock are a must and should not be neglected.

## 5. Conclusions

1. Polish beekeepers have been following recommendations on queen bee replacement.
2. A large percentage of Polish beekeepers regularly replaced and purchased queens, and this is related to apiary size and location.
3. The involvement of purchased queens in colony management is associated with the decade, the size of the apiary, and its location.
4. The percentage of purchased queens significantly increases with the number of colonies in the apiary (this tendency is displayed by apiaries with no more than 80 colonies).
5. In order to maintain such a trend and further improve Polish apiaries, it is necessary to continue educational efforts and research in this area.

## Figures and Tables

**Table 1:** Number of questionnaires by decade timeslot and by apiary size and geographical location.

TIMESLOT	← APIARY SIZE	Cd * →	C	C	C	C	C	N	N	S	S	W	W	SW	SW	SW	E	E	IN TOTAL
		VOIVODESHIP ↓	Kujawsko-pomorskie	Łódzkie	Mazowieckie	Świętokrzyskie	Wielkopolskie	Pomorskie	Warmińsko-mazurskie	Małopolskie	Podkarpackie	Lubuskie	Zachodniopomorskie	Dolnośląskie	Opolskie	Śląskie	Lubelskie	Podlaskie	
**1980–1989**	**1–10**		15	10	21	7	10	0	0	23	10	10	2	4	3	7	8	0	**132**
	**11–20**		8	25	16	7	22	6	17	20	21	13	8	7	9	26	6	1	**215**
	**21–50**		25	38	15	2	64	40	34	43	38	19	54	49	15	58	51	27	**572**
	**51–80**		20	19	11	0	42	23	19	11	24	19	31	23	2	28	22	10	**304**
	**>80**		17	8	0	0	20	12	11	5	11	0	8	7	2	4	7	12	**124**
**1990–1999**	**1–10**		7	16	5	0	2	11	2	17	5	0	2	0	0	11	2	14	**94**
	**11–20**		29	21	1	6	6	1	6	2	4	4	10	9	4	19	11	8	**141**
	**21–50**		49	24	2	7	12	28	24	31	30	15	22	22	2	27	32	24	**351**
	**51–80**		17	5	4	1	11	7	8	10	9	21	8	4	0	17	4	9	**135**
	**>80**		9	9	4	0	8	8	6	3	12	0	15	1	0	2	5	11	**93**
**2000–2009**	**1–10**		7	9	6	0	0	9	0	0	0	2	1	0	0	4	2	3	**43**
	**11–20**		5	7	25	1	4	2	5	0	3	0	2	5	0	10	11	5	**86**
	**21–50**		12	20	20	11	12	1	5	16	14	17	5	8	0	34	21	18	**214**
	**51–80**		20	5	10	2	4	1	0	17	7	6	5	3	0	11	8	2	**101**
	**>80**		10	3	1	0	3	9	0	0	8	3	15	0	0	2	0	2	**56**
**2010–2018**	**1–10**		2	5	1	0	0	7	0	3	4	2	2	0	0	0	2	5	**34**
	**11–20**		7	2	10	7	3	4	0	12	5	0	1	1	0	0	6	2	**60**
	**21–50**		4	11	5	5	17	5	1	17	1	10	12	2	3	1	20	14	**128**
	**51–80**		5	1	5	0	8	5	0	14	0	2	3	1	4	0	6	2	**56**
	**>80**		2	0	1	0	1	0	0	6	0	3	9	0	1	0	2	0	**25**

* Cardinal directions on the map of Poland: C = central, N = north, S = south, W = west, SW = southwest, and E = east.

**Table 2:** Probability of *Wald* χ^2^ test statistic for the tested classification for beekeepers and their interaction in generalized linear models (GLMs) estimated for investigated dependent variables.

Effect	Beekeepers	
	Replacing Queens	Purchasing Queens
**Decade (De)**	0.575	0.200
**Apiary size (As)**	0.000	0.000
**Voivodeship (Vo)**	0.000	0.000
**De × As**	0.432	0.000
**De × Vo**	0.000	0.117
**As × Vo**	0.000	0.000

**Table 3:** Percentage of queens replaced by beekeepers, analyzed by apiary size and calculated on the basis of the data from 1980 to 2018.

Apiary Size	Percentage of Replaced Queens (X)			
	X = 0%	0% < X ≤ 50%	50% < X < 100%	X = 100%
**1–10**	21.5	37.6	25.7	15.2
**11–20**	8.6	39.5	46.0	5.9
**21–50**	4.8	39.1	53.3	2.8
**51–80**	5.0	44.5	49.3	1.2
**>80**	6.7	43.3	48.7	1.3
**mean**	7.4	40.5	48.0	4.1

**Table 4:** Probability of *Wald χ*^2^ test statistic for the tested classification for queens and their interaction in GLMs estimated for investigated dependent variables.

Effect	Queens	
	Replaced	Purchased
**Decade (De)**	0.990	0.005
**Apiary size (As)**	0.000	0.000
**Voivodeship (Vo)**	0.999	0.000
**De × As**	0.000	0.000
**De × Vo**	0.000	0.000
**As × Vo**	0.000	0.000

## Appendix A

**Table A1:** Numerical characteristics of beekeepers replacing queens in their apiaries, including those who introduced purchased queens, analyzed by decade timeslot and apiary size.

Timeslot	Apiary Size	Beekeepers Replacing Queens		Beekeepers Purchasing Queens	
		n	(%)	n	(%)
**1980–1989**	**1–10**	106	80.3	49	37.1
	**11–20**	195	90.7	100	46.5
	**21–50**	550	96.2	288	50.3
	**51–80**	286	94.1	167	54.9
	**>80**	121	97.6	69	55.6
	**total/average**	**1258**	**93.4**	**673**	**50.0**
**1990–1999**	**1–10**	77	81.9	31	33.0
	**11–20**	129	91.5	55	38.3
	**21–50**	333	94.9	180	51.3
	**51–80**	127	94.1	67	49.6
	**>80**	86	92.5	64	68.8
	**total/average**	**753**	**92.4**	**396**	**48.6**
**2000–2009**	**1–10**	33	76.7	17	39.5
	**11–20**	75	87.1	53	62.4
	**21–50**	201	93.9	153	71.5
	**51–80**	99	98.0	73	72.3
	**>80**	50	89.3	39	69.4
	**total/average**	**457**	**91.6**	**335**	**67.1**
**2010–2018**	**1–10**	25	73.5	12	35.3
	**11–20**	58	96.7	36	60.0
	**21–50**	122	93.3	94	73.4
	**51–80**	55	98.2	43	76.8
	**>80**	22	88.0	16	64.0
	**total/average**	**282**	**93.1**	**201**	**66.3**
**in total**		**2750**	**92.8**	**1066**	**54.2**

**Table A2:** Percentage of replaced queens by beekeepers, analyzed by decade timeslot and apiary size.

Timeslot	Apiary Size	Percentage of Replaced Queens (X)			
		X = 0%	0% < X ≤ 50%	50% < X < 100%	X = 100%
**1980–1989**	**1–10**	22.2	35.7	26.3	15.8
	**11–20**	8.0	32.5	51.0	8.4
	**21–50**	5.6	36.9	54.3	3.2
	**51–80**	4.1	43.2	51.4	1.4
	**>80**	9.8	34.5	54.6	1.1
	**average**	8.0	36.9	50.3	4.9
**1990–1999**	**1–10**	18.1	34.0	33.0	14.9
	**11–20**	8.5	23.4	59.6	8.5
	**21–50**	5.1	31.6	59.0	4.3
	**51–80**	6.7	47.4	45.2	0.7
	**>80**	8.6	33.3	55.9	2.2
	**average**	7.9	33.4	53.4	5.4
**2000–2009**	**1–10**	27.9	32.6	14.0	25.6
	**11–20**	10.6	36.5	43.5	9.4
	**21–50**	7.0	38.8	52.3	1.9
	**51–80**	2.0	36.6	59.4	2.0
	**>80**	10.7	35.7	53.6	0.0
	**average**	8.8	37.1	49.1	5.0
**2010–2018**	**1–10**	26.5	44.1	23.5	5.9
	**11–20**	3.3	48.3	41.7	6.7
	**21–50**	4.7	48.4	44.5	2.3
	**51–80**	1.8	44.6	51.8	1.8
	**>80**	12.0	36.0	52.0	0.0
	**average**	7.6	46.9	42.9	2.6

**Table A3:** Numerical characteristics of replaced queens, including purchased ones, analyzed by decade timeslot and apiary size.

Timeslot	Apiary Size	Replaced Queens		Purchased Queens	
		N	(%)	N	(%)
**1980–1989**	**1–10**	132	46.5	132	21.6
	**11–20**	215	47.9	215	20.5
	**21–50**	572	52.2	572	13.7
	**51–80**	304	48.2	304	10.5
	**>80**	124	49.1	124	4.8
	**total/average**	**1347**	**49.8**	**1347**	**14.0**
**1990–1999**	**1–10**	94	51.3	92	34
	**11–20**	141	60.0	137	20
	**21–50**	351	58.0	349	21.5
	**51–80**	135	47.3	135	11
	**>80**	93	53.0	93	22
	**total/average**	**815**	**55.2**	**807**	**21.0**
**2000–2009**	**1–10**	43	47.09	43	21.8
	**11–20**	85	53.3	85	35.5
	**21–50**	214	52.2	214	32.2
	**51–80**	101	53.9	101	30.7
	**>80**	56	51.3	56	26.7
	**total/average**	**499**	**52.2**	**499**	**30.9**
**2010–2018**	**1–10**	34	35.6	34	24.9
	**11–20**	60	51.9	60	32.5
	**21–50**	128	51.3	128	36.5
	**51–80**	56	54.0	56	35.5
	**>80**	25	49.5	25	16.5
	**total/average**	**303**	**50.0**	**303**	**32.6**
**in total**		**2964**	**51.7**	**2956**	**20.8**

**Table A4:** Summary numerical characteristics of replaced queens, including purchased ones, analyzed only by apiary size (from 1980 to 2018).

Apiary Size. (No. of Colonies)	Replaced Queens		Purchased Queens	
	N	(%)	N	(%)
**1–10**	303	47.0	301	25.8
**11–20**	501	52.7	497	24.4
**21–50**	1265	53.7	1263	21.3
**51–80**	596	49.5	596	16.4
**>80**	298	50.7	298	15.3
**in total**	**2963**	**51.7**	**2955**	**20.7**

## References

[1] The Beekeeping Sector in Poland in 2019. http://www.inhort.pl/files/program_wieloletni/PW_2015_2020_IO/spr_2019/Semkiw_2019_Sektor_pszczelarski_zad.4.3.pdf.

[2] Bieńkowska M., Widle J., Panasiuk B., Gerula D. Bee breeding activity in Poland. Proceedings of the SICAMM 2018 Conference.

[3] Hatjina F., Bieńkowska M., Charistos L., Chlebo R., Costa C., Drazic M.M., Filipi J., Gregorc A., Ivanova E.N., Kezić N. (2014). A review of methods used in some European countries for assessing the quality of honey bee queens through their physical characters and the performance of their colonies. J. Apic. Res..

[4] Büchler R., Costa C., Hatjina F., Andonov S., Meixner M.D., Le Conte Y., Uzunov A., Berg S., Bieńkowska M., Bouga M. (2014). The influence of genetic origin and its interaction with environmental effects on the survival of *Apis mellifera* L. colonies in Europe. J. Apic. Res..

[5] Gray A., Brodschneider R., Adjlane N., Ballis A., Brusbardis V., Charrire J.D., Chlebo R., Coffey M.F., Cornelissen B., da Costa C.A. (2019). Loss rates of honey bee colonies during winter 2017/18 in 36 countries participating in the COLOSS survey, including effects of forage sources. J. Apic. Res..

[6] Van Der Zee R., Brodschneider R., Brusbardis V., Charrière J.-D., Chlebo R., Coffey M.F., Dahle B., Dražić M.M., Kauko L., Kretavičius J. (2014). Results of international standardised beekeeper surveys of colony losses for winter 2012–2013: Analysis of winter loss rates and mixed effects modelling of risk factors for winter loss. J. Apic. Res..

[7] Sperandio G., Simonetto A., Carnesecchi E., Costa C., Hatjina F., Tosi S., Gilioli G. (2019). Beekeeping and honey bee colony health: A review and conceptualization of beekeeping management practices implemented in Europe. Sci. Total. Environ..

[8] Akyol E., Yeninar H., Korkmaz A., Cakmak I. (2008). An observation study on the effects of queen age on some characteristics of honey bee colonies. Ital. J. Anim. Sci..

[9] Plate M., Bernstein R., Hoppe A., Bienefeld K. (2019). The importance of controlled mating in honeybee breeding. Genet. Sel. Evol..

[10] Bixby M., Baylis K., Hoover S.E., Currie R.W., Melathopoulos A., Pernal S.F., Foster L.J., Guarna M.M. (2017). A Bio-Economic Case Study of Canadian Honey Bee (Hymenoptera: Apidae) Colonies: Marker-Assisted Selection (MAS) in Queen Breeding Affects Beekeeper Profits. J. Econ. Èntomol..

[11] Giacobino A., Molineri A., Cagnolo N.B., Merke J., Orellano E., Bertozzi E., Masciangelo G., Pietronave H., Pacini A., Salto C. (2016). Queen replacement: The key to prevent winter colony losses in Argentina. J. Apic. Res..

[12] Lee K.V., Goblirsch M., McDermott E., Tarpy D.R., Spivak M. (2019). Is the Brood Pattern within a Honey Bee Colony a Reliable Indicator of Queen Quality?. Insects.

[13] Tarpy D.R., Keller J.J., Caren J.R., Delaney D.A. (2012). Assessing the Mating ‘Health’ of Commercial Honey Bee Queens. J. Econ. Èntomol..

[14] Bieńkowska M. (2017). Instrumental Insemination of Honey Bees—New Knowledge and Practice.

[15] Bieńkowska M. (2017). The Importance of the Queen Bee in a Colony. Personal communication.

[16] Bieńkowska M. (2016). New Trends in Bee Breeding and Apiary Production. The most Effective Methods of Introduction of Queen Bees, before Oviposition, to Colonies. Personal communication.

[17] English: Honeybee queen management in Poland. Proceedings of the XLIX Naukowa Konferencja Pszczelarska.

[18] SmartBees—Sustainable Management of Resilient Bee Populations. http://www.smartbees-fp7.eu/.

[19] EurBeST—European Honey Bee Breeding and Selection Team. https://eurbest.eu/.

[20] Gromisz M. (1975). Replacement of honey bee queens in Polish apiaries. Pszczel. Zesz. Nauk..

[21] Porporato M., Grillone G., Patetta A., Manino A., Laurino D. (2015). Survey of the Health Status of Some Honey Bee Queens in Italy. J. Apic. Sci..

[22] Mutinelli F. (2011). The spread of pathogens through trade in honey bees and their products (including queen bees and semen): Overview and recent developments. Rev. Sci. et Tech. de l’OIE.

[23] Muñoz I., Pinto M.A., De La Rúa P. (2014). Effects of queen importation on the genetic diversity of Macaronesian island honey bee populations (*Apis mellifera* Linneaus 1758). J. Apic. Res..

[24] Meixner M.D., Francis R.M., Gajda A., Kryger P., Andonov S., Uzunov A., Topolska G., Costa C., Amiri E., Berg S. (2014). Occurrence of parasites and pathogens in honey bee colonies used in a European genotype-environment interactions experiment. J. Apic. Res..

[25] Șeker İ., Köseman A., Karlıdağ S., Aygen S. (2017). Beekeeping activities II: The evaluation of beekeeping activities in terms of beekeeper preferences, production quality and bee diseases in Malatya Province. JOTAF.

[26] Chauzat M.-P., Cauquil L., Roy L., Franco S., Hendrikx P., Ribière-Chabert M. (2013). Demographics of the European Apicultural Industry. PLoS ONE.

[27] Döke M.A., McGrady C.M., Otieno M., Grozinger C.M., Frazier M. (2018). Colony Size, Rather Than Geographic Origin of Stocks, Predicts Overwintering Success in Honey Bees (Hymenoptera: Apidae) in the Northeastern United States. J. Econ. Èntomol..

[28] De La Rúa P., Jaffe R., Dall’Olio R., Muñoz I., Serrano J. (2009). Biodiversity, conservation and current threats to European honeybees. Apidologie.

[29] Pérez-Sato J.A., Âline N.C., Martin S.J., Hughes W.O.H., Ratnieks F.L.W. (2009). Multi-level selection for hygienic behaviour in honeybees. Heredity.

[30] Krajowy Rejestr Sądowy. http://www.krs-online.com.pl/msig-2101-4099.html.

